# A novel approach to simultaneously scan genes at fragile sites

**DOI:** 10.1186/1471-2407-6-205

**Published:** 2006-08-08

**Authors:** Pascale Willem, Jacqueline Brown, Jan Schouten

**Affiliations:** 1 Department of Hematology and Molecular Medicine University of the Witwatersrand and the National Health Laboratory Services, WITS Medical School, 8 York road, 2193 Parktown, South Africa; 2 MRC Holland, Hudsonstraat, Amsterdam, The Netherlands

## Abstract

**Background:**

Fragile sites are regions of the genome sensitive to replication stress and to exposure to environmental carcinogens. The two most commonly expressed fragile sites FRA3B and FRA16D host the histidine triad (*FHIT*) and WW domain containing oxidoreductase (*WWOX*) genes respectively. There is growing evidence that both genes contribute to cancer development and they are frequently altered by allelic and homozygous deletions in a variety of tumors. Their status is linked to prognosis in several malignancies and they are thought to be involved in early tumorigenesis.

The loci for *FHIT *and *WWOX *both span over a megabase but the genes encode for small transcripts. Thus the screening of intragenic deletion can be difficult and has relied on loss of heterozygosity LOH assays, or genomic arrays.

**Methods:**

Multiplex ligation dependent probe amplification MLPA, allows for the detection of deletions/duplications and relative quantification of up to 40 specific probes in a single assay. A *FHIT/WWOX *MLPA assay was designed, applied and validated in five esophageal squamous cell carcinoma ESCC, cell lines established in South Africa where this cancer is of high prevalence. Sixteen probes covered all *FHIT *exons and 7 probes covered *WWOX*.

**Results:**

Both homozygous and hemizygous deletions were detected in *FHIT*, in four of the cell lines with a preferential deletion of exons 5 and 4. Chromosome 3 short arm was present in normal copy number indicating that deletions were site specific. In contrast *WWOX *was not altered in any cell lines. RT-PCR expression pattern paralleled the pattern of deletions. Ten primary ESCC tumor specimens were subsequently screened with this assay. *FHIT *exon deletions were found in four of them.

**Conclusion:**

This method offers an alternative to loss of heterozygosity studies. Simultaneous scanning of *FHIT *and *WWOX *exons in the context of early tumorigenesis and tumor progression, may help clarify the mechanistic events related to cancer development which are not revealed by imuno histochemistry assays. The presence of site specific deletions of *FHIT *in these cell lines and primary tumors support its possible role in South African ESCC and justifies a wider screening.

## Background

The two most commonly expressed human fragile sites, FRA3B and FRA16D, harbor genes which have a tumor suppressive function, namely the fragile histidine triad, *FHIT*, and the WW domain containing oxidoreductase, *WWOX *genes respectively [1-3]. Both genes have small exons distributed over their respective fragile loci and large intragenic deletions have been detected in a wide variety of malignant and pre-malignant tumors, reviewed in [4,5]. In particular, *FHIT *inactivation can occur in early stages of carcinogenesis [6], and also correlates significantly with malignant progression [7-9]. Both *WWOX *and *FHIT *have been implicated in cancers that are strongly associated with environmental carcinogens such as smoking and alcohol consumption.

Esophageal squamous cell carcinoma ESCC is a common cancer in South Africa, ranking as second most common malignancy in black males and third most common in black females [10]. Similarly to other parts of the world where this cancer is of high prevalence, a strong association between environmental exposure and the risk of developing ESCC has been demonstrated. Risk factors include heavy smoking [11], exposure to fumonisin, a fungal toxin produced by fusarium fungi growing on local maize [12], consumption of alcohol, and home made beer fermented from infected maize [13,14]. Human Papilloma virus HPV infection, and poor nutrition, have been made to make a contribution to the development of ESCC [15].

The above risk factors and carcinogens have the potential to affect the integrity of fragile sites directly or indirectly. Tobacco exposure increases the expression of common fragile sites [16], HPV preferentially integrates within fragile sites loci [17], alcohol and fumonisin both affect folate intake which may facilitate the expression of fragile sites [18-20], and fumonisin exposure in cell cultures increases the incidence of chromosomal damage [21,22]. This strengthens the hypothesis that the combinatorial effect of all or some of these factors may have a role in the initiation of genetic instability early in the disease and that fragile sites may be early targets. At present the genes within the most common fragile sites *FHIT *and *WWOX *have not been examined in South African ESCC.

The most commonly used method to investigate deletions in *FHIT *and *WWOX *is loss of heterozygosity (LOH) analysis in separate assays [23-25]. Other methods such as immuno histochemical (IHC) assays have provided a considerable amount of data regarding the loss of Fhit protein in a variety of cancers [26-29], IHC however does not inform on the nature of inactivating genetic events that may reflect the role of etiological factors. RT-PCR studies are dependent on the availability of fresh material, which are often difficult to obtain, as most biopsy specimens are very small or fixed in paraffin. Since genomic deletions and hypermethylation appear to be the main mechanism of *FHIT *and *WWOX *inactivation [6,9,30,31] a significant amount of information can be retrieved from the retrospective evaluation of archived paraffin embedded specimens.

Although the *FHIT *locus spans 1,67 Mb and *WWOX *spans more than 750 kb [2,32], both genes only code for transcripts of around 1 kb. It should be noted that the detection of LOH in the respective fragile locus does not always reflect the loss of coding exons or of protein product [25]. In addition, deletions within fragile sites may be heterogeneous within one cell line, which could bias LOH results.

In order to obtain an exon specific, cost effective and high throughput method to screen ESCC specimens for *FHIT *and *WWOX *genomic deletions, a new assay was designed using the existing multiplex ligation-dependent probe amplification MLPA technology [33]. This assay allowed for the detection of deletions/duplications and relative quantification of both *FHIT *and *WWOX *exons in a single run. We evaluated its performance investigating *FHIT *and *WWOX *deletions in five ESCC South African established cell lines. The assay was subsequently used to screen the genomic status of these two genes in ten primary ESCC tumors.

## Methods

### Cell lines

The five esophageal carcinoma cell lines investigated here were originally established from black South African patients with known esophageal squamous cell carcinoma. These were previously described in the literature and referred to as cell lines: SNO, WHCO1, WHCO3, WHCO5 and WHCO6, [34,35]. Cells were grown in Dulbecco Modified Eagles medium (DMEM): HAMS F12 (GIBCO) (3:1) supplemented with 10% fetal calf serum (FCS) in a humidified atmosphere at 37°C.

### ESCC primary tumor tissue samples

Endoscopic esophageal tumor biopsies were collected from ESCC patients by a gastroenterologist in the course of routine diagnostic investigations. Touch preparations were prepared from each sample for histological assessment and were examined by an experienced pathologist.

The research was approved by the University of the Witwatersrand Ethical Committee and patients were enrolled after written consent was obtained.

### DNA preparation

After trypsinisation and centrifugation of the cultured ESCC cells, DNA was extracted by standard phenol:chloroform extraction followed by ethanol precipitation. DNA from tumor biopsies was extracted by the same method.

### RT-PCR

RNA was extracted from each cell line using the Qiagen RNeasy kit (Qiagen GmbH, Hilden Germany) according to the manufacturer's instructions. RT-PCR for the *FHIT *full transcript was performed using previously published primer sequences [36]. Primers were designed for *WWOX *full transcript analysis. They were: forward primer 5'-GAG TTC CTG AGC GAG TGG-3' mapping in exon 1, and reverse: 5'-GCT CGT TGG AGA AGA GGA-3' mapping in exon 9.

### Fluorescence *in-situ *hybridization, FISH

Metaphases from each cell line were prepared as per standard cytogenetic techniques. FISH analysis using probes specific for the short arm of chromosome 3 and the long arm of chromosome 16 (Qbiogene, Strasbourg, France) was performed as per manufacturer's protocols (Universal FISH protocol).

### MLPA

The MLPA method initially developed and described by Schouten *et al*, [33] was used to detect exons specific copy number change in the *FHIT *and *WWOX *genes. Briefly, a series of MLPA probes each consisting of 2 target specific hemi probes, one of which is linked to a stuffer sequence of variable length, and with two end sequences recognized by universal MLPA primer pair are hybridized to the test DNA. Once hybridized to the target DNA the 2 hemi probes, designed next to each other, can be ligated and PCR amplified. The stuffer sequence allows for each specific probe set amplification product to have a defined size and to be separated by capillary electrophoresis. For each probe the amount of PCR product obtained reflects the amount of target DNA in the sample and relative target copy number can be measured.

Sixteen probes for the *FHIT *gene covering all 10 exons (two probes set covering exons 1 to 5 as well as exon 8) and 7 probes for the *WWOX *gene covering 5 out of 9 exons were developed. Fifteen other probes for other human genes on chromosome 3p and 16q as well as other chromosomes, (8p, 9q, 10p, 12p, 16p), were included as internal control.

The MLPA reaction was performed as previously described [33] using ± 200 ng of DNA from each cell line and primary tumor.

### Experimental data analysis

The PCR amplified products were separated by capillary electrophoresis on an ABI 3100 Genetic Analyzer (Applied Biosystems, Foster City, CA, USA). The peak heights values were obtained by Gene Scan analysis software (Applied Biosystems) and exported to an excel spreadsheet for further processing.

Ten DNA samples extracted from healthy individuals were first analyzed to assess the pattern of probe amplification in normal tissue, to create a mean "reference control" pattern, and to exclude the possibility of copy number polymorphism. For each probe in each sample run, the relative peak height was calculated by dividing its absolute value by the sum of all probes peak height for this run. A mean normalized peak height was calculated for each probe and standard deviation established. All five ESCC tumor cell lines were then processed in duplicate from cell cultures grown at different time and with differing DNA extractions. External control samples were included in each run. The cell lines amplification patterns were consistent across runs. The ratio of each cell line probe peaks to the control sample probe peaks was first examined using relative peak height values.

Since a large number of chromosomal abnormalities may be present in cancer cells, the normalization of probe peaks by relative values (percentage) can distort the overall profile of probe amplification. Normalization to the ratio of the absolute peak height of 'control probes' in the tumor by the peak height of 'control probes' in the external reference DNA is therefore preferable and has been used with differing MLPA probes set [37,38]. Assigning this ratio a value of one, it can be applied to normalize all peaks in a run. The choice of adequate control probes is important due to the number of genetic alterations that may be present in cancer cells and affect "control probes" themselves. Here we have used an absolute peak ratio, selecting internal control probes whose pattern of amplification did not deviate from that of the reference control in the same run as seen on the original Gene Scan data.

## Results

### Amplification pattern in controls

**Figure 1:**
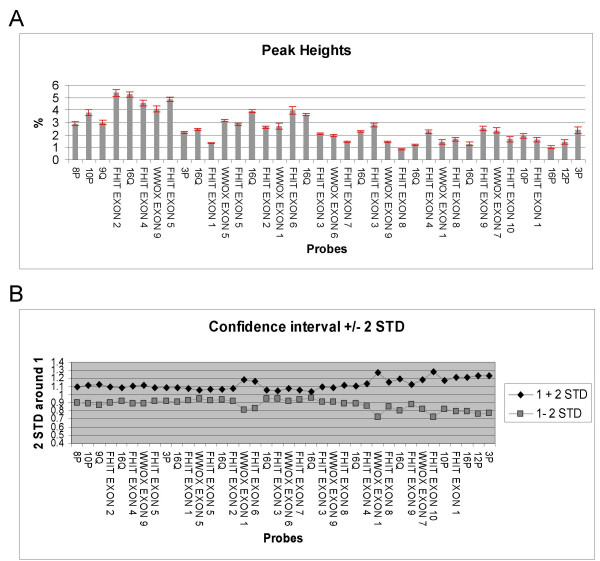
**(A) **Mean "reference control" pattern of probe amplification established from 10 normal individual samples. Each probe is represented on the horizontal axis in the order of their size. The normalized peak heights are expressed in percentages on the vertical axis. **(B) **Two standard deviations above and below the mean peak height (normalized to one) for each probe are shown and validate the scoring of deletion/amplification within the: 0.7–1.3 ranges. Standard deviations are generally bigger for larger probes.

Five males and 5 female control DNA samples were used to establish the relative pattern of probe amplification in normal samples. A mean reference control pattern was derived (380 probe points) (Figure 1A). Although sample size was small, standard deviations were used based on previous MLPA results and the consistency of amplification patterns [39]. This was done to verify the recommended confidence interval for interpretation of deletion and amplifications in this assay. In the mean reference control, when each probe peak value was normalized to one, the addition of two standards deviations above and below respectively remained within a range of 1.3 and 0.7 (Figure 1B). A ratio of less than 0.7 was considered as a deletion and a ratio greater than 1.3 was considered as a gain.

### Comparison of *FHIT *and *WWOX *DNA copy number in cell lines by MLPA and FISH

**Figure 2:**
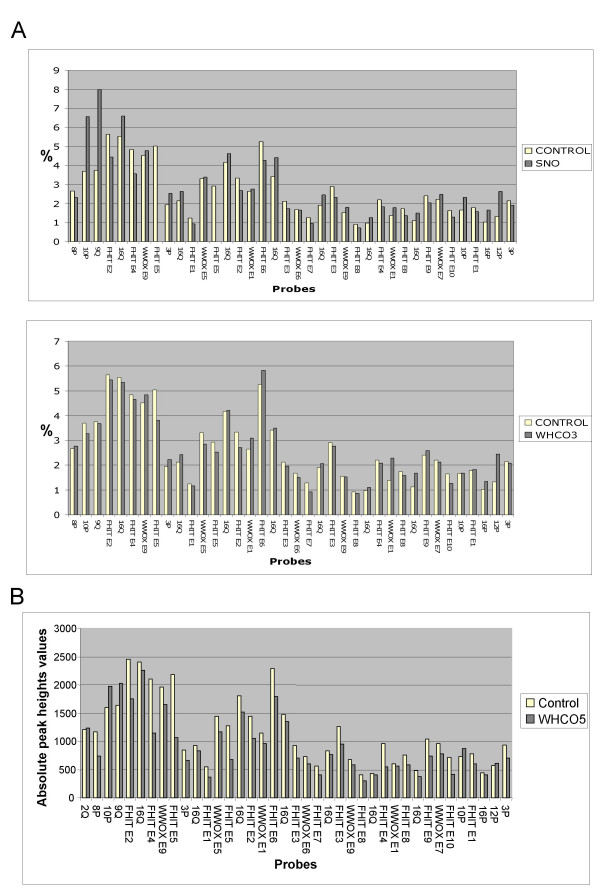
**(A) Comparison of relative peak height values: control/cell lines**. Comparisons of relative probe peak heights between cancer cell lines in dark grey, and control, in yellow. *FHIT *exon specific probes are marked *FHIT *E1, E2...and *WWOX *exon probes: *WWOX *E1, E2. Internal controls probes are labeled according to their chromosomal location. **Top**, cell line SNO showing a homozygous deletion of *FHIT *exon 5 with both exon 5 probes. The second and third internal control probes, on 10p and 9q, appeared increased in copy number in SNO. They were not used to normalize data when ratios were derived from absolute peak height values. **Middle**, no significant deletion nor amplification in either *FHIT *or *WWOX *was seen in WHCO3. **(B) Absolute peak height values, comparison control/cell line WHCO5**. **Bottom**, comparison of absolute probe peak heights between WHCO5 in dark grey, and control, in yellow. *FHIT *exons 4 and 5 were present in half copy number. Controls probes on chromosome 16 were consistent with the external control.

For subsequent experiments using the tumor cell lines, control specimens were included in the run and experiments were done in duplicate. First the relative peak height value for each probe, in each cell line, was compared to the same probe relative peak value in the external control (Figure 2A). Cell line WHCO3 had a probe amplification profile comparable to the control with no significant changes in *FHIT *and *WWOX *(Figure 2A). A complete homozygous deletion of *FHIT *exon 5 was detected in cell line SNO while the other *FHIT *exons were under represented. Relatives (not shown) and absolute peak heights comparison of tumor to control specimen, showed *FHIT *exons 4 and 5 deletion in cell line WHCO5 (ratio of 0.55) (Figure 2B), compatible with a hemizygous deletion.

**Figure 3:**
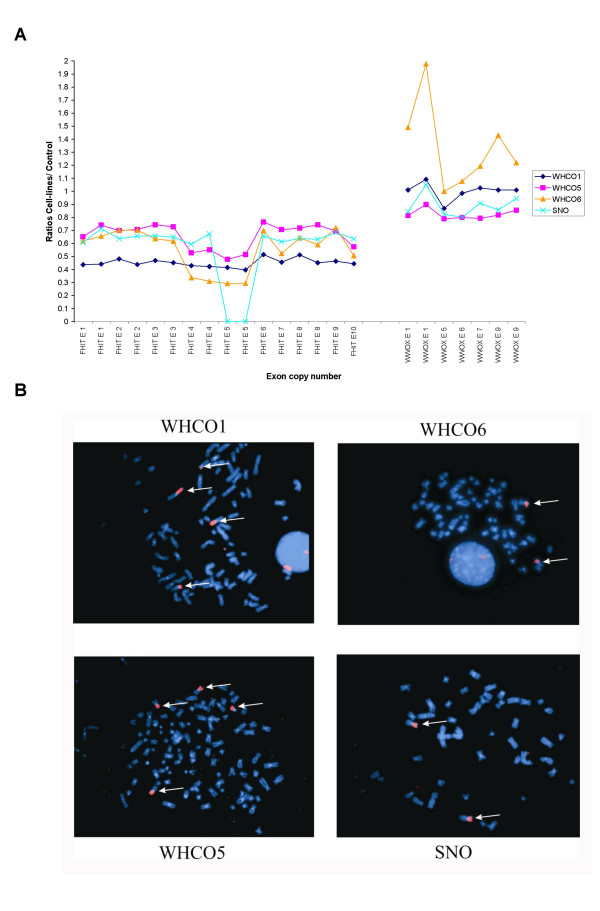
**(A) **Graphic representation of *FHIT *and *WWOX *exon copy number in each cell line. Absolute ratios were normalized to a control probe whose amplification was consistent to that of the external control sample. Exons in their coding order (horizontal axis) have been represented. **(B) **FISH with chromosome 3 short arm specificprobe, 3p, labeled with a red fluorochrome (arrowed) in each cellline.

Because some internal control probes appeared to be increased in the tumor cell lines, we normalized the peak height ratios to a control probe that did not deviate from the same probe in the external control sample of the same run, (original Gene Scan data). The results are summarized in Figure 3A, which shows *FHIT *and *WWOX *exons copy number in each cell line. The ratios derived from absolute values were comparable to ratios seen with relative values (Figure 2), but clarified the level of significance of deletions and corrected the level of some control probe over representation.

As for cell line WHCO5, WHCO6 had a preferential deletion of *FHIT *exons 4 and 5, in addition all *FHIT *exons in this cell line had a ratio near or below 0.7 compatible with a hemizygous deletion of the full gene. Cell line WHCO1 had a homogeneous *FHIT *deletion across all exons consistent with hemizygous deletion. In both cell lines delineated as SNO and WHCO5, the internal control probes that mapped near the FRA3B locus, at 3p25 and 3p22 respectively were not deleted, although cell line SNO had a homozygous deletion of *FHIT *exon 5. *FHIT *deletions in these cell lines were specifically confined to the FRA3B region. In cell line WHCO1 the probe mapping at 3p22 was deleted, putting the telomeric boundary of the deletion between 3p25 and 3p22. In cell line WHCO6, which had a preferential deletion of exons 4 and 5 of the *FHIT *gene, both 3p probes were underrepresented compatible with hemizygous deletion of this region. However FISH using a chromosome 3 short arm library 3p, showed 2 copies in this cell line (Figure 3B and Table 1). Both cell lines SNO and WHCO1 had 2 or more 3p copies (Figure 3B and Table 1). Cell line WHCO5 had a chromosome number of 117 with 4 to 5 copies of most chromosomes and four copies of 3p. MLPA does not detect balanced copy number changes such as tetraploidies. The unbalanced deletion of 3p was clearly detected by MLPA. In all cell lines the 3p deletion was therefore specific for the region surrounding FRA3B.

By contrast in none of the cell lines *WWOX *deletions could be detected, with all ratios having values above 1 (but below 1.3). Cell line WHCO6 showed an increased copy number for exon 1 and 9; chromosome 16 q was however present in 3 to 4 copies in this cell line shown by FISH analysis with a chromosome 16q library, (results not shown). This was paralleled by an increased MLPA signal for some of the control probes mapping to 16q and included in this assay.

### RT-PCR

**Figure 4:**
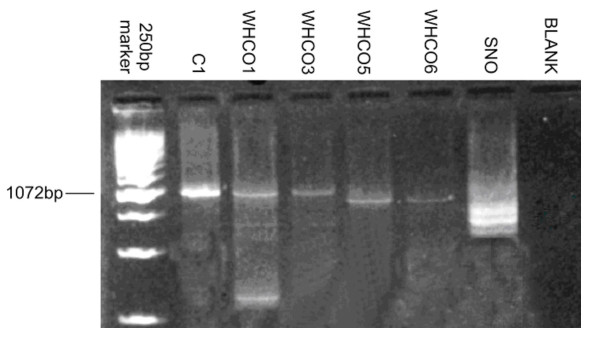
A 2% agarose gel, showing RT-PCR results of *FHIT *expression (full transcript) investigated in the 5 ESCC cell lines.

All cell lines having a deletion in one or more exons of *FHIT*, had an aberrant *FHIT *RT-PCR pattern (Figure 4). Both cell lines WHCO5 and 6 showed the presence of a smaller transcript that could represent alternative splicing of the remaining allele. Cell line WHCO1 showed a weak full transcript as well as an additional small product. Cell line SNO exhibited 2 aberrant size products on RT-PCR that were consistent across experiments. Sequencing however showed non-coding transcripts, probably due to the fact that exon 5 is the first *FHIT *coding exon. RT-PCR specific for exon 5 confirmed the deletion in cell line SNO (results not shown). Cell line WHCO3, which did not exhibit any deletion, had a normal *FHIT *transcript although it seemed weakly amplified.

RT-PCR on *WWOX *showed a normal amplification product size in all cell lines (results not shown). The combined information of MLPA, FISH and RT-PCR is summarized in Table 1.

### Evaluation of *FHIT *and *WWOX *DNA copy number in primary tumors by MLPA

**Figure 5:**
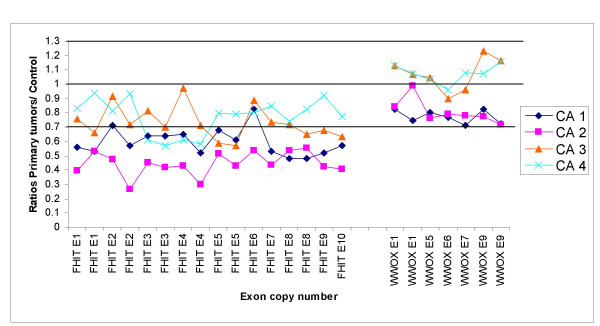
Graphic representation of *FHIT *and *WWOX *exon copy number in four primary ESCC tumors. Absolute peak ratios were normalized to control probes not affected in the tumor, (10p, 8p). Exons in their coding order (horizontal axis) have been represented.

Ten ESCC primary tumors were investigated with the MLPA assay. Four controls were included in the reaction, and MLPA reactions were performed in duplicate. Six tumor specimens showed an amplification profile comparable to that of the mean reference control with no significant exon deletions of *FHIT *or *WWOX*, (results not shown). Four tumor specimens showed deletions spanning the *FHIT *locus. Figure 5 shows the respective absolute peak height ratios of tumor samples to control, for *FHIT *and *WWOX *exon specific probes. ESCC samples delineated as CA1 and CA2 showed a pattern consistent with a hemizygous deletion of the whole *FHIT *locus; a preferential deletion of exons 7 to 10 was noticeable in CA1 (Figure 5) and included the probe at 3p22. The deletion extended over 3p22 and 3p25 in the tumor delineated as CA2. The tumor specimens delineated as CA3 and CA4 had preferential deletions of *FHIT *specific exons, *FHIT *exon 5, and *FHIT *exons 3 and 4 respectively (Figure 5). As observed in the permanent ESCC cell lines, *WWOX *exons were not deleted in these primary tumors.

## Discussion

This study describes the value of an innovative approach using an MLPA assay specifically designed to scan the exon intragenic composition of the *FHIT *and *WWOX *genes involved at fragile sites, here evaluated in the setting of ESCC South African cell lines and primary tumor specimens.

Many studies have used LOH, assays to detect large intragenic deletion in genes at fragile sites [40-42]. However, deletions occurring within fragile site are themselves heterogeneous [1,43], they can be discontinuous and do not always affect coding exons [32]. In this approach, the detection of deletions is reflected by ratios (Figure 3) and does not depend on the presence of informative markers. Hemi and homozygous genomic deletions encompassing *FHIT *and *WWOX *exons were targeted to reflect the exon dosage composition of the genes. The internal control probe used to normalize the peak heights should be selected in each cancer sample and should have an amplification pattern consistent with its equivalent in control samples included in the same run. Fifteen internal control probes dispersed in the genome expanded the possibilities.

In this study there was a correlation between the presence of *FHIT *exon deletions and aberrant pattern of RNA expression in the cell lines. *FHIT *expression was generally very low in the established ESCC cell lines. Due to its exon based design, this assay would miss intronic genomic deletions that do not affect exon integrity but may inhibit *FHIT *expression as was observed in some studies [32]. In this regard it is worth noting that since most of the *FHIT/WWOX *probes map within exons, the assay could be used for RNA studies.

Despite some controversy regarding the involvement of *FHIT *in oncogenesis there is mounting evidence to suggest that it is more than a passive actor, although the nature of its role in differing cancer scenarios subtypes remains to be elucidated.* FHIT *is altered in a wide variety of tumors, mostly by genomic deletions and/or promoter hypermethylation, which results in inhibition of the *FHIT *product. Several reports have raised pertinent questions regarding the relevance of *FHIT *deletion to cancer development: exon skipping alternative transcripts have been found in normal tissues in addition to normal transcripts [44-46], the gene lies within an instable genomic region which may be mechanistically deleted and the effect of hemizygous deletion in tumors is unclear. Experimental models have shown that *FHIT *full or haplo insufficiency confers an increased sensitivity to carcinogen exposure [47]. *FHIT *has an anti-apoptotic activity [48] and it is possible that *FHIT *decreased expression facilitates the occurrence of added deleterious genetic events.

Four of five SA ESCC cell lines and four of ten primary tumors had a *FHIT *deletion suggesting that *FHIT *might be of relevance in SA esophageal cancer as found in other parts of the world where the incidence of this type of cancer is high [7,31]. Exons 4 and 5 tended to be the preferential target in three cell lines and two primary tumors, as in previous observations [1,49]. Cell line SNO had a homozygous deletion of exon 5 and no functional transcript. The other three cell lines had hemizygous deletion of *FHIT *exons, with an abnormal expression pattern. The evaluation of chromosome 3p copy number by FISH as well as the internal control probes on 3p in MLPA established that these deletions were specific for the FRA3B region in ESCC cell lines and in three of four primary tumors. Two of the primary tumors, had an MPLA profile consistent with hemizygous deletion of the *FHIT *locus. *FHIT *has previously been investigated in South African oral squamous cell carcinoma (OSCC) [50,51] which is known to share similar etiological risk factors with ESCC. In one study, the authors found reduced or absent Fhit protein in 12 of 17 tumors and aberrant RT-PCR products in a third of the cases [50]. In the present study, *FHIT *deletions were associated with aberrant transcripts in the cell lines and deletions were observed in a third of primary ESCC tumors. It would be necessary to screen a larger cohort of primary tumors to evaluate the importance of *FHIT *and *WWOX *intragenic deletions in SA ESCC.

In contrast to *FHIT*, no deletion was observed in *WWOX *in either the cell lines or the primary tumors. Some studies have shown that the simultaneous loss of expression of *FHIT *and *WWOX *is frequent in several cancers [52-54]. Since all common fragile sites are sensitive to external carcinogens these findings raise questions as to the exact mechanism involved in these cell lines. *FHIT *loci could have been altered early in the disease process and a clone be driven/facilitated by its loss with FRA 3B being the most sensitive fragile site [55]. Deletions could also represent a later event where *FHIT *haplo-insufficiency aggravated an already unstable genetic background. *WWOX *might be concordantly inactivated via a differing mechanism. While LOH at the *WWOX *locus has been reported in ESCC [56], *WWOX *is frequently inactivated by promoter hypermethylation [57,58], which was not detected in this assay.

*FHIT *loss, with or without *WWOX *loss, has been shown to correlate positively with tumor invasiveness and prognosis in several cancers (breast, pancreas, gastric cancers) [54,59,60]. At the same time, Fhit protein loss can be detected early in some cancer, including ESCC, and appears to correlate with the stages of malignant transformation [7]. Whether involved early in the disease or deleted as a consequence of genomic instability and enrolled as a partner in the progression of the malignant phenotype it will be important to establish the role of genes at fragile sites in relation to specific carcinogen exposure and disease behavior. Interestingly two recent and independents studies have shown that both in patients and experimentally induced pre-malignant lesions there is an activation of the DNA damage response and increased genetic instability at fragile sites, before genomic instability [61,62]. The detection of genetic imbalances at fragile sites may therefore be of value to detect the "signature" of replication stress [63] and may assist to characterize pre-malignant lesions.

## Conclusion

The *FHIT/WWOX *assay described in this study may offer a rapid and cost effective method to assist a deletion screening of tissue lesions associated with heavy carcinogen exposure, (esophageal, head and neck, and lung pathologies). It may help clarify whether genes at fragile sites tend to be concordantly deleted.

In this study, the assay detected *FHIT *deletions in four of five SA ESCC cell lines and four of ten primary tumors, which justifies a wider screening in SA ESCC patients. The *FHIT/WWOX *MLPA kit is likely to be a valuable tool in other cancer where its prognostic and early screening value will have to be assessed further.

## Competing interests

J Schouten has a patent on MLPA technology and owns MRC Holland.

## Authors' contributions

P W conceived the study carried out MLPA and FISH assays and drafted this manuscript. J B did the RT_PCR studies. J S designed and developed the MLPA specific probes. All authors read and approved the final manuscript.

## Pre-publication history

The pre-publication history for this paper can be accessed here:



## Figures and Tables

**Table 1:** Summary of FISH, MLPA and RT-PCR results for FHIT on the five ESCC cell lines.

**Cell lines**	**Modal chromosome number**	FISH 3p copy number*	**MLPA *FHIT *exon dosage deletion**	**RT-PCR**
Control	46	2	Normal	Normal transcript
SNO	47	2+	Homozygous deletion exon 5	Aberrant transcripts only
WHCO1	52	2+ 2 small	Hemizygous deletion exons: 1 to 10	Normal transcript and weak expression Aberrant transcript
WHCO3	51	2+	Normal	Normal transcript Weak expression
WHCO5	117	4	Hemizygous deletion exons 4 and 5	Aberrant transcript only and weak expression
WHCO6	47	2	Hemizygous deletions exons 1, 3, 4, 5, 7, 10. (preferential 4 and 5)	Aberrant transcript only and weak expression

*: A sign + in the FISH column refers to additional 3p copy number full or partial.
